# Add in a virus: four cases of severe Kawasaki disease and concurrent adenovirus infection

**DOI:** 10.1186/s12887-025-05982-1

**Published:** 2025-10-02

**Authors:** Michael J. Harrison, Leah Githinji, Claire Butters, Georgia Dewey, Zinzile Ngwenyama, George Comitis, Heloise Buys, Liesl Zühlke, Brian Eley, Diana Hardie, Kate Webb

**Affiliations:** 1 Department of Paediatrics and Child Health, University of Cape Town, Cape Town, South Africa; 2 Department of Paediatrics, Nelson Mandela University, Gqeberha, South Africa; 3 Division of Paediatric Rheumatology, Department of Paediatrics and Child Health, University of Cape Town, Cape Town, South Africa; 4 Department of Paediatrics and Child Health, Dora Nginza Hospital, Gqeberha, South Africa; 5 Walter Sisulu University, Gqeberha, South Africa; 6 Western Cape Paediatric Cardiology Service, Red Cross War Memorial Children’s Hospital, Cape Town, South Africa; 7 Ambulatory & Emergency Paediatrics, Red Cross War Memorial Children’s Hospital, Cape Town, South Africa; 8 South African Medical Research Council, Francie Van Zujl Drive Parow, Cape Town, South Africa; 9 Paediatric Infectious Diseases Unit, Red Cross War Memorial Children’s Hospital, Cape Town, South Africa; 10 Division of Medical Virology, University of Cape Town, Cape Town, South Africa; 11 Crick African Network, Francis Crick Institute, London, United Kingdom

**Keywords:** Kawasaki disease, Viral infections, Adenovirus, Hyperinflammatory syndrome, Macrophage activation syndrome, Immunological triggers

## Abstract

**Background:**

Kawasaki disease is an idiopathic systemic vasculitis which predominantly occurs in young children. Approximately one third of children with Kawasaki disease have a concurrent acute infection. Several cases of severe and complicated Kawasaki disease in the setting of concurrent adenovirus infection have been described in the literature.

**Case presentations:**

Four children, between the ages of 9 months and 2 ½ years, presented to two centres in South Africa between October 2023 and March 2024 with simultaneous adenovirus infection and Kawasaki disease. All four cases fulfilled American Heart Association 2017 diagnostic criteria for typical Kawasaki disease. Adenovirus infection was confirmed by polymerase chain reaction testing of nasopharyngeal aspirate specimens and, in one case, was further confirmed on pleural fluid analysis. A unifying feature of these four cases was marked severity of Kawasaki disease. All four cases were complicated by macrophage activation syndrome. Two patients exhibited IVIG resistance, defined by recrudescent fever more than 36 h after initial IVIG therapy, and two children developed coronary artery abnormalities. These children were primarily managed with IVIG therapy. Two patients received multiple IVIG doses due to IVIG resistance. All four patients received adjuvant steroid therapy, which was indicated for macrophage activation syndrome. All four children were discharged after several weeks. Disease resolution was confirmed at follow up in three of four cases; one patient was lost to follow up.

**Conclusions:**

These cases are illustrative of the challenges of distinguishing between acute infections and Kawasaki disease, and managing cases with concurrent infection. We postulate that adenovirus infection may trigger immune dysregulation in at-risk children, resulting in a hyperinflammatory syndrome which is clinically consistent with Kawasaki disease and macrophage activation syndrome.

## Background

Kawasaki disease (KD) is an idiopathic systemic vasculitis. KD typically presents in children between the ages of six months and five years as an acute illness characterized by fever, conjunctivitis, rash, cervical lymphadenopathy, peri-oral erythema, and extremity swelling. These features often follow a brief prodrome of respiratory or gastrointestinal illness and may not all be present at the same point in time.

Recent population-based time series analyses have demonstrated a correlation between seasonal viral infections and KD incidence [1, 2]. One third of children with KD have a concurrent acute infection, [3] and approximately 41% of KD patients have a respiratory virus detected on nasopharyngeal polymerase chain reaction (PCR) testing [4].

The differential diagnosis in a child with KD is broad, as KD shares common features with many childhood infectious conditions, such as streptococcal infections and viral exanthems (e.g. measles, adenovirus, Epstein-Barr virus), rheumatic disorders (e.g. systemic juvenile idiopathic arthritis) and drug reactions (e.g. Stevens-Johnson syndrome) [5]. Distinguishing between acute infections and KD early in the illness course is often challenging, particularly when concurrent infection is present. Diagnostic confusion between KD and acute infections contributes to delays in instituting early immunomodulatory management, which may contribute to higher complication rates.

The treatment of KD involves intravenous immunoglobulin (IVIG) and sometimes steroids [6]. These treatments are not used in viral infections. The decision to use these therapies in resource-limited settings is further complicated by cost, particularly of IVIG.

KD is a self-limiting condition. Without therapy, the features of acute inflammation resolve after an average of 12 days [7]. Early IVIG therapy is associated with a significant reduction in complications, particularly of coronary artery aneurysms (CAA), which are reported in 25% of untreated cases as opposed to 3–5% of treated KD cases [6, 8]. A smaller subset of KD cases (1–2%) may complicate with macrophage activation syndrome (MAS) [9, 10]. MAS is a life-threatening syndrome of immune hyperactivation, characterized by cytopenias, liver dysfunction, coagulopathy, and encephalopathy, and is associated with a high mortality rate (8–22%) [11, 12]. The key positive prognostic factor for children with MAS is early diagnosis and treatment with high-dose steroid therapy, and sometimes other immunosuppressive agents. Significant overlap in the clinical presentation of KD and MAS makes it challenging to distinguish between the two disorders. A high index of suspicion is required for prompt detection of MAS in children with KD, which is critical to facilitate early treatment and improve outcomes.

We report a cluster of cases of concurrent human adenovirus (HAdV) infection and KD complicated by MAS presenting between October 2023 and March 2024 at the Red Cross War Memorial Children’s Hospital in Cape Town, South Africa and Dora Nginza Hospital in Gqeberha, South Africa. Ethical approval for this case series was obtained from the University of Cape Town Human Research Ethics Committee (*HREC 314/2024*).

## Case presentations

### Case 1 (Fig. 1)

**Fig. 1:**
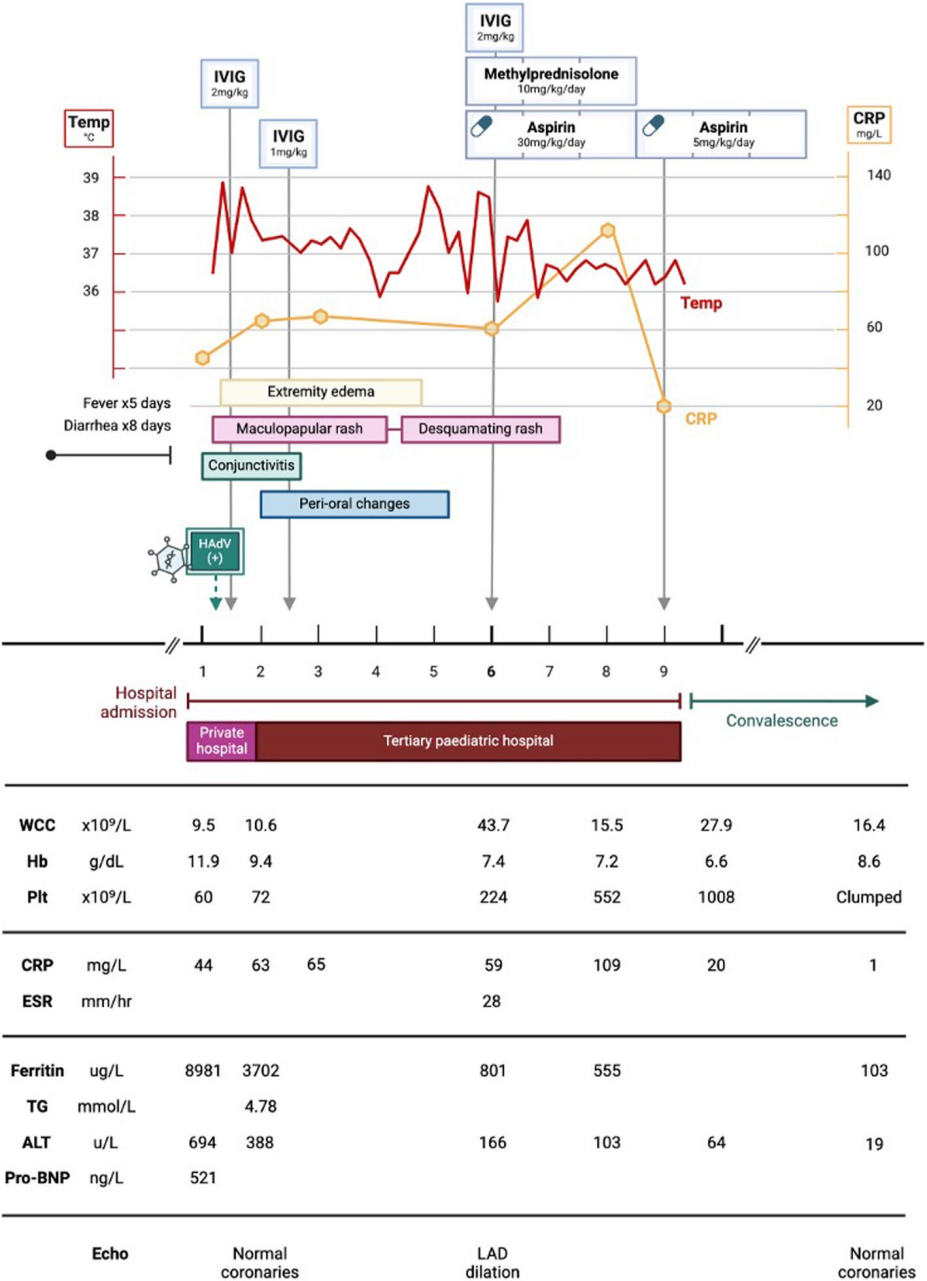
Case 1, clinical and laboratory features. IVIG (intravenous immune globulin); HAdV (human adenovirus); WCC (white cell count); Hb (haemoglobin); Plt (platelet count); CRP (C-reactive protein); ESR (erythrocyte sedimentation rate); TG (triglycerides); ALT (alanine transaminase); pro-BNP (pro-brain natriuretic peptide); LAD (left anterior descending coronary artery)

A nine-month-old boy presented to his paediatrician with an eight-day history of diarrhoea. He had no significant medical history, normal growth and development, and was fully immunised, including Bacillus Calmette-Guérin (BCG). He was admitted for management of dehydration and acute kidney injury, which responded well to intravenous fluid therapy. On the second day of admission he developed high-grade fever, rash, and multiorgan dysfunction (encephalopathy, transaminitis, bicytopenia, and coagulopathy). He was started on empiric intravenous ceftriaxone for suspected sepsis and was transferred to a paediatric referral hospital for further assessment.

Clinical examination revealed an acutely ill child with pallor, cervical lymphadenopathy, and oedematous hands and feet. He was tachycardic and pyrexial, with normal perfusion and blood pressure. An erythematous, maculopapular rash was noted over the trunk, axillae, and limbs. Nonexudative conjunctivitis and red, fissured lips were present. He was lethargic, with no meningism or focal signs. Abdominal palpation revealed hepatomegaly, but no splenomegaly. Laboratory investigations (Fig. 1) demonstrated hyperferritinaemia, bicytopenia, transaminitis, hypofibrinogenaemia, elevated D-dimer, and hypertriglyceridaemia, which prompted concern of MAS. Nasopharyngeal aspirate PCR was HAdV-positive; cycle threshold (Ct) value was not available at retrospective investigation. Urine culture revealed extended-spectrum beta lactamase (*ESBL) Klebsiella pneumoniae* and *Candida albicans*. In light of clear urine dipsticks and polymicrobial growth, this likely represented contamination. Further work-up is summarised in Table 1. Echocardiography on the first day of admission demonstrated normal function and normal coronary vessels.

**Table 1:** Ancillary investigations (Cases 1–4)

	**Case 1**	**Case 2**	**Case 3**	**Case 4**
HIV serology	Negative	Negative	Negative	Negative
SARS-CoV2 antigen	Negative	Negative	Negative	Negative
SARS-CoV2 antibody	Negative	Positive	Negative	Negative
Blood culture	Negative	Negative	Negative	Negative
Urine culture	*K. pneumoniae* (contaminant)	Negative	Negative	Negative
Stool culture	-	Negative	Negative	-
Tuberculosis work-up	-	Negative	Negative	Negative
CSF analysis	-	-	Normal	Normal
BMAT	-	-	-	-

*HIV* (Human Immunodeficiency Virus), *SARS-CoV2* (severe acute respiratory syndrome related to coronavirus-2), *CSF* (cerebrospinal fluid), *BMAT* (bone marrow aspirate/trephine)

There was significant debate over the diagnosis of KD versus adenoviral infection. Ultimately, the child was assessed to have KD complicated by MAS, with concurrent HAdV infection. Differential diagnoses of bacterial sepsis and multisystem inflammatory syndrome in children (MIS-C) were considered. He was admitted to the high-dependency unit and treated with IVIG 2 g/kg over 24 h on the first day of admission and broad-spectrum antibiotic therapy (meropenem). Supportive management included supplemental oxygen, vitamin K and furosemide. On the second day of admission ongoing fever, persistent organ dysfunction and rising serum C-reactive protein (CRP) prompted administration of a second dose of IVIG 1 g/kg, infused over 48 h due to fluid overload. This was followed by marked improvement in clinical condition and laboratory parameters, with defervescence, resolution of encephalopathy, and reduction in serum ferritin, transaminases, and CRP.

**Fig. 2:**
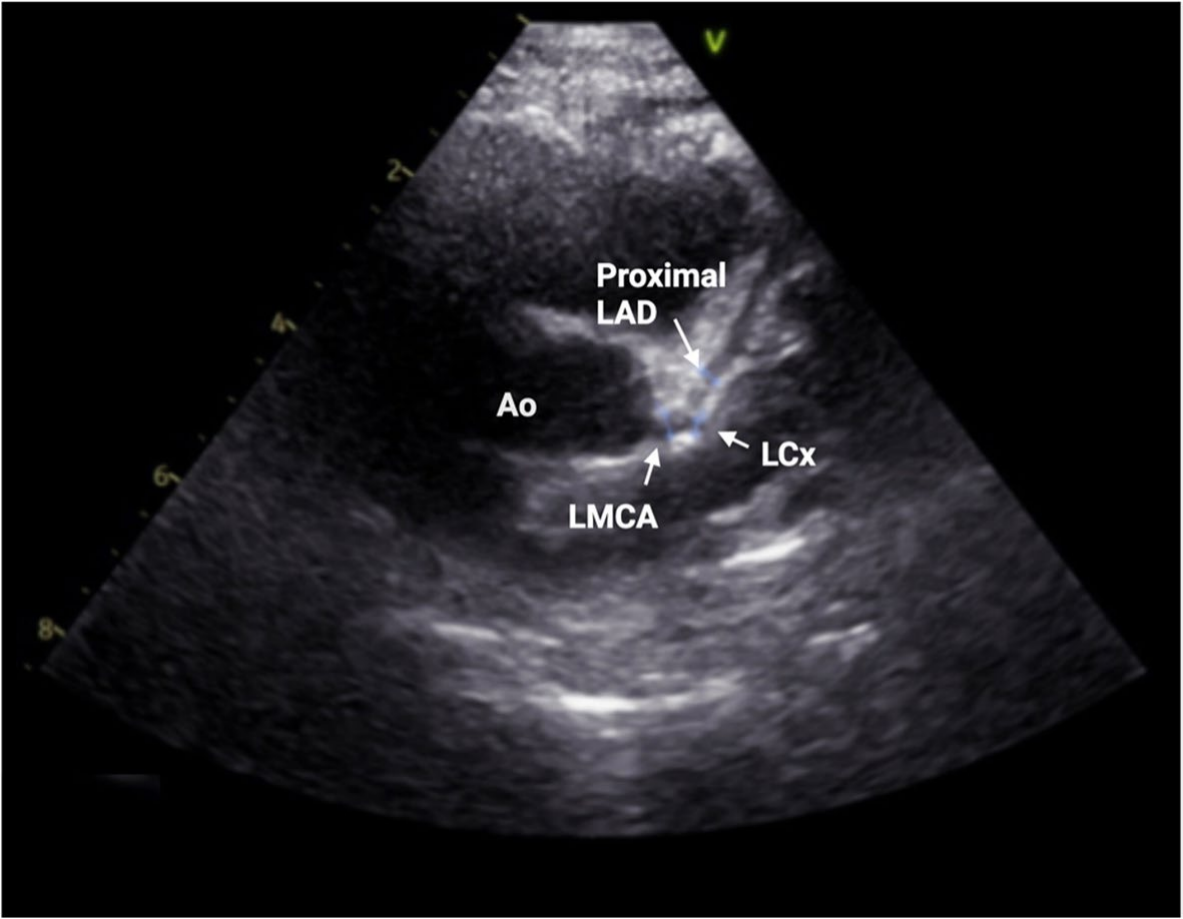
Case 1, echocardiogram on day 6 of admission. Parasternal short axis view, demonstrating proximal LAD dilatation (diameter 2.3 mm; z-score 3.8). The LMCA (diameter 2.88 mm; z-score 1.64) and proximal left circumflex artery (diameter 1.65 mm) are normal. — Ao (Aorta); LAD (left anterior descending artery); LMCA (left main coronary artery); LCx (left circumflex artery)

On the sixth day of admission he developed new temperature spikes, rising CRP trend, and evidence of CAA, signifying refractory KD. However, platelet count, transaminases, and ferritin continued to improve, suggesting resolution of MAS. Echocardiogram on the sixth day of admission demonstrated a left anterior descending CAA(diameter 2.3 mm; Z-score 3.8) (Fig. 2). Data from the Paediatric Heart Network Normal Echocardiogram Database was used to estimate coronary artery z-scores throughout this paper [13]. He was treated with another dose of IVIG 2 g/kg over 12 h, aspirin 30 mg/kg/day and once daily intravenous methylprednisolone 10 mg/kg for three consecutive days. Fever resolved over the following 24 h. He continued to improve clinically and was discharged after 10 days in hospital on aspirin 5 mg/kg/day. At follow up six weeks later, he was clinically well with normal coronary arteries on echocardiography, and aspirin therapy was discontinued.

### Case 2 (Fig. 3)

**Fig. 3:**
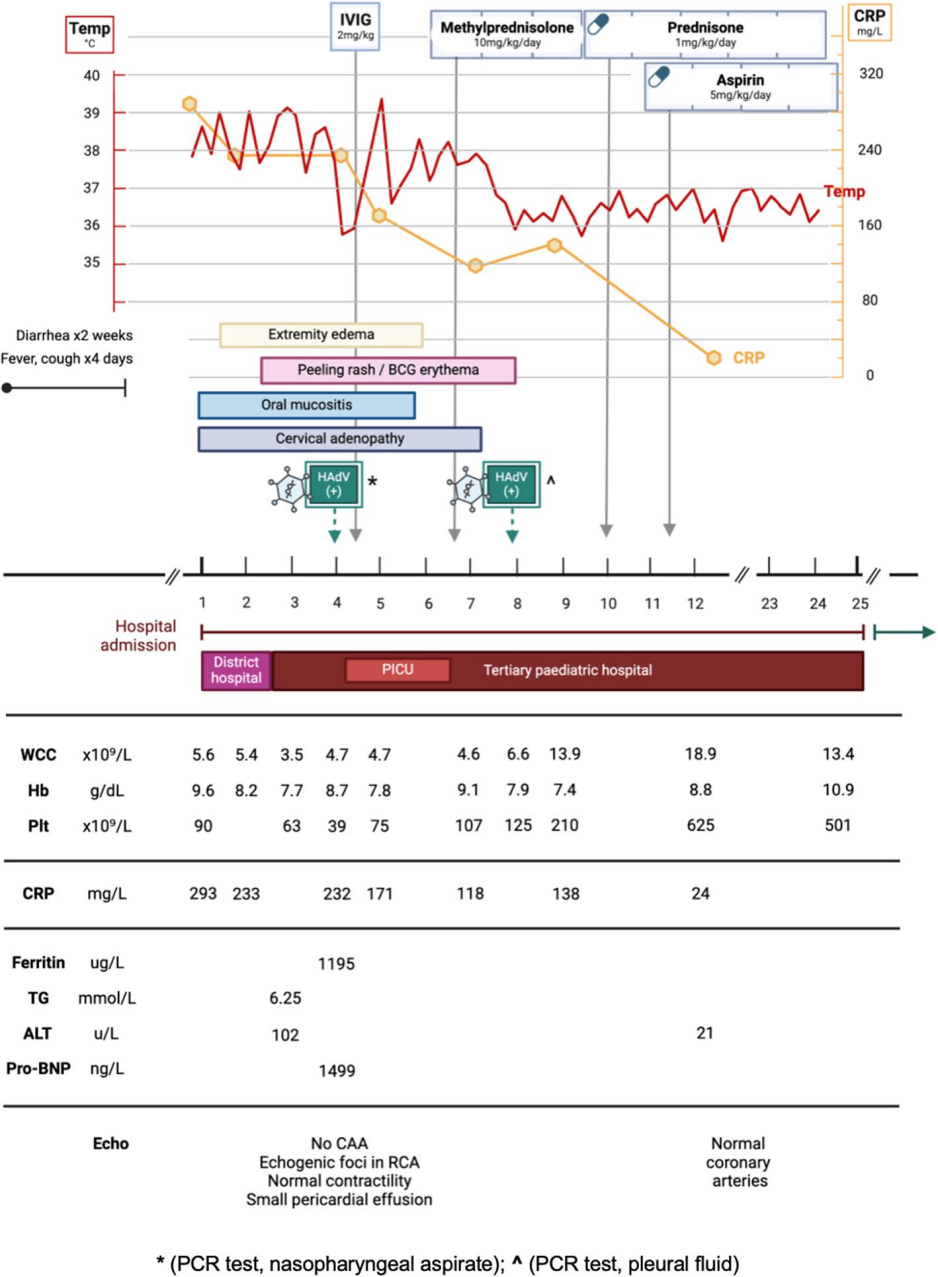
Case 2, clinical and laboratory features. IVIG (intravenous immune globulin); HAdV (human adenovirus); BCG (Bacillus Calmette-Guerin); PICU (paediatric intensive care unit); WCC (white cell count); Hb (haemoglobin); Plt (platelet count); CRP (C-reactive protein); TG (triglycerides); ALT (alanine transaminase); pro-BNP (pro-brain natriuretic peptide); CAA (coronary artery aneurysm); RCA (right coronary artery)

**Fig. 4:**
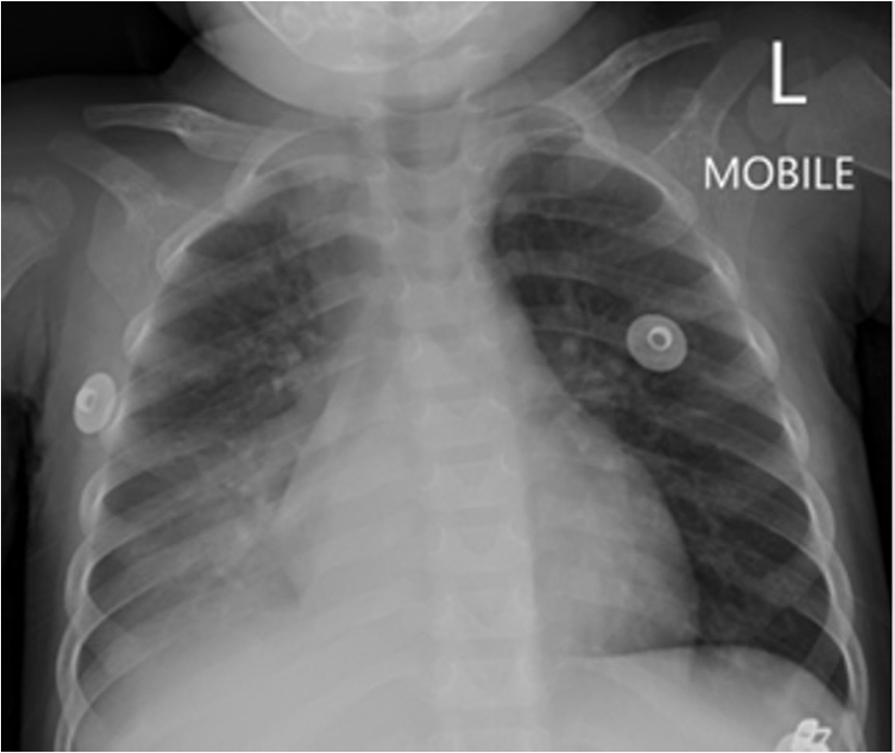
Case 2, chest radiograph on day 1 of admission. Chest radiograph (anterior–posterior projection) showing air-space consolidation in the right middle lobe and right lower lobe, with segmental atelectasis of the right upper lobe

A 19-month-old girl presented with a two-week history of gastroenteritis and four-day history of cough and fever. She was growing well, with no past medical history. She had completed the South African national immunisation schedule up until age nine months, including BCG, but had since missed two immunisations (measles second dose, hexavalent DTaP-HBV-IPV-Hib fourth dose) due at 12 and 18 months, respectively. She was admitted to a district hospital with a diagnosis of community-acquired pneumonia and gastroenteritis for supplemental nasal prong oxygen, intravenous rehydration fluids, and intravenous antibiotic therapy (amoxicillin/clavulanic acid). On the second day of admission she developed progressive respiratory distress, high-output watery diarrhoea and high-grade fever. Chest radiograph demonstrated right middle and lower lobe consolidation (Fig. 4). Antibiotic therapy was escalated to intravenous ceftriaxone. She was started on continuous positive airway pressure (CPAP) support and was transferred to a paediatric referral hospital.

On clinical examination she appeared acutely unwell, with pallor, lymphadenopathy, oral mucositis, and generalised oedema. She was tachycardic and pyrexial, with normal blood pressure. On CPAP she remained tachypnoeic with subcostal recessions and a fraction of inspired oxygen (FiO2) requirement of 0.6. Auscultation revealed coarse crepitations and bronchial breathing across the right hemithorax. She was irritable, without meningism or focal neurological signs. Palpation of the abdomen demonstrated hepatomegaly, but no splenomegaly. Musculoskeletal examination was normal.

Laboratory investigations (Fig. 3) were notable for pancytopenia, hyperferritinaemia and elevated CRP. Nasopharyngeal aspirate PCR was HAdV-positive, with Ct value of 14.95, suggesting acute infection. Further work-up is summarised in Table 1.

The child was initially diagnosed with adenoviral gastroenteritis and pneumonia. The differential diagnoses included KD and MIS-C. She was admitted to the high-dependency unit on CPAP, empiric antibiotic therapy, and intravenous fluids. However, her clinical condition continued to deteriorate, with persistent fever, metabolic acidosis, respiratory distress and worsening pancytopenia. A desquamating rash was noted over the trunk, with erythema at the BCG scar. On day 4 of admission she was transferred to the intensive care unit (ICU) and received a first dose of IVIG 2 g/kg over 12 h.

**Fig. 5:**
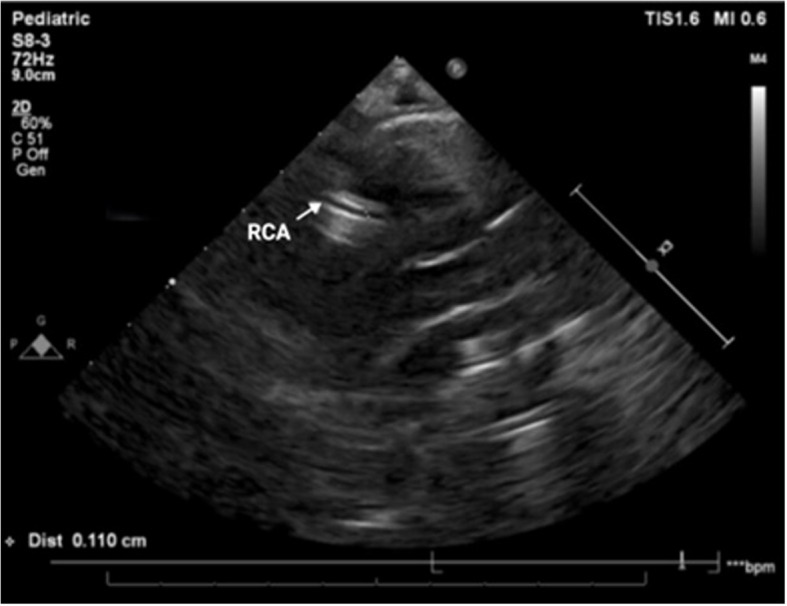
Case 2, echocardiogram on day 4 of admission. Parasternal short axis view, demonstrating hyperechoic walls of RCA, which is not dilated (diameter 1.66 mm; z-score 0.08). — RCA (right coronary artery)

Echocardiography demonstrated normal calibre coronary arteries, with hyperechoic foci in the right coronary artery, normal myocardial contractility, and a small pericardial effusion (Fig. 5).

**Fig. 6:**
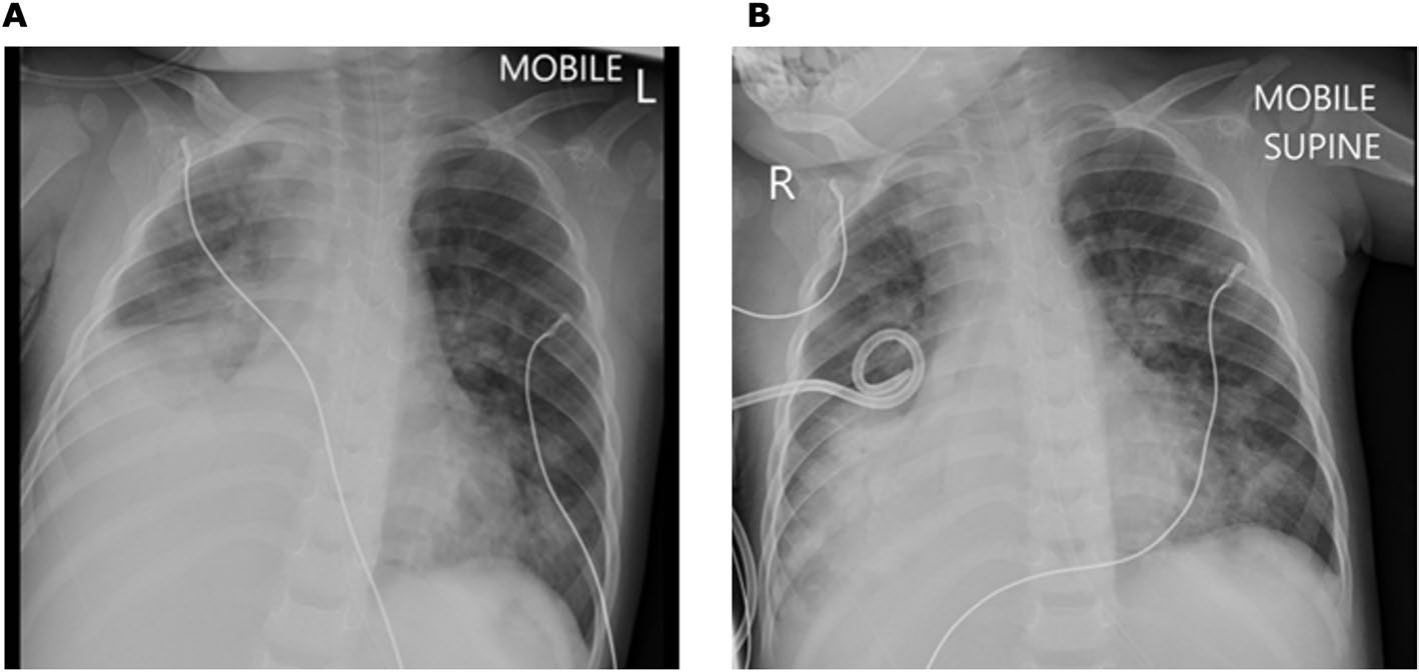
Case 2, Chest radiographs on day 4 of admission. **A** Chest radiograph (anterior–posterior projection) showing right lower zone opacification with blunting of the costophrenic angle suggestive of right pleural effusion, with compression atelectasis of right middle lobe and right lower lobe; associated opacification in right upper lobe, left lingula, and left lower lobe. **B** Chest radiograph (anterior–posterior projection) showing right intercostal pigtail drain in situ with partial drainage of pleural effusion; associated dense residual homogeneous opacity in the right lower lobe and multilobar infiltrates involving the right middle lobe, left lingula, and left lower lobe

The child was assessed to have KD considering persistent fever, lymphadenopathy, desquamating rash, oral mucositis, extremity oedema, and BCG reactivation. This was complicated by MAS, as evidenced by hyperferritinaemia, hypertriglyceridaemia, pancytopenia, lethargy, and liver dysfunction. Chest radiograph and ultrasound demonstrated right parapneumonic pleural effusion, which was subsequently drained via pigtail thoracostomy drain (Fig. 6). Pleural fluid analysis demonstrated an exudate with lymphocyte predominance; HAdV was detected on pleural fluid PCR (Ct value 29.83); microscopy and culture were negative. Intravenous methylprednisolone was administered at 10 mg/kg once daily for three consecutive days, followed by prednisone 1 mg/kg/day orally, tapered over a week. The steroid pulse was followed by defervescence, normalising CRP trend, and gradual resolution of the cytopenias. She also received aspirin 5 mg/kg/day. Repeat echocardiography on day 14 of admission showed no coronary abnormalities.

The child was transferred from ICU back to the ward. The admission was further complicated by persistent gastroenteritis due to acquired lactase deficiency and nosocomial infection which resolved on empiric piperacillin-tazobactam and amikacin therapy for five days. Blood and urine cultures were negative. She was discharged home in a stable condition after 25 days in hospital. Unfortunately, she did not return for scheduled follow up visits.

### Case 3 (Fig. 7)

**Fig. 7:**
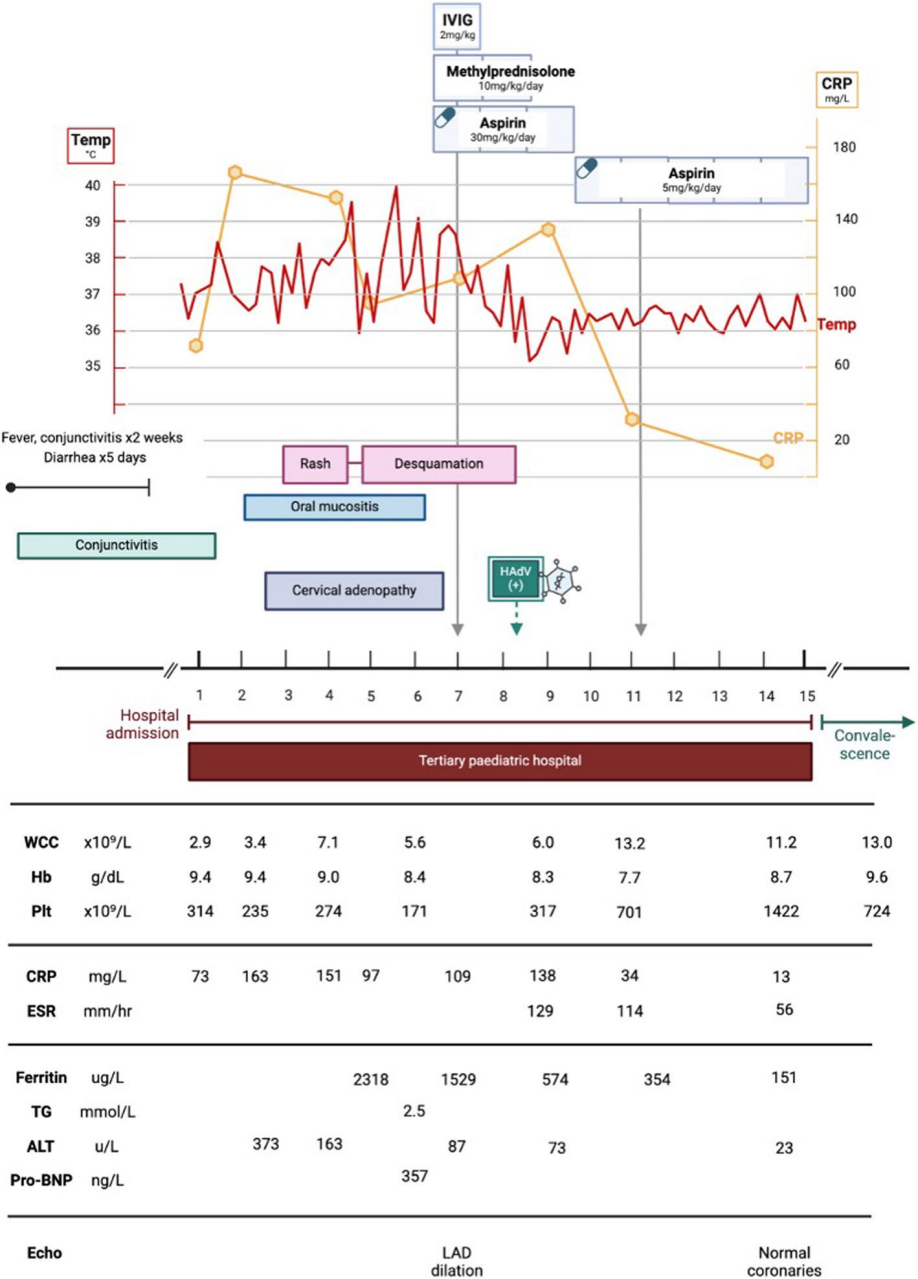
Case 3, Clinical and laboratory features. IVIG (intravenous immune globulin); HAdV (human adenovirus); WCC (white cell count); Hb (haemoglobin); Plt (platelet count); CRP (C-reactive protein); ESR (erythrocyte sedimentation rate); TG (triglycerides); ALT (alanine transaminase); pro-BNP (pro-brain natriuretic peptide); LAD (left anterior descending coronary artery)

A 22-month-old boy presented with a two-week history of fever, red eyes and cough, with associated diarrhoea for five days. He had been seen at his primary care clinic on two occasions during the illness episode and received paracetamol and amoxicillin, without improvement. He then presented directly to a paediatric referral hospital. He had BCG, according to the local schedule.

Clinical examination demonstrated an unwell, lethargic child with dehydration, tachycardia and fever. On initial examination, there were no rashes, palpable lymph nodes, or peri-oral changes; however, over the following six days he developed cervical lymphadenopathy, oral mucositis and an erythematous desquamating perineal rash. He was tachypnoeic, with bilateral crepitations on auscultation of his chest. There was no hepatosplenomegaly, and musculoskeletal examination was unremarkable. Initial chest radiograph was unremarkable. Laboratory investigations (Fig. 7) were notable for bicytopenia, hyperferritinaemia, and elevated CRP. Nasopharyngeal aspirate PCR was HAdV-positive, with a Ct value of 19.47, compatible with acute infection. Further work-up is summarised in Table 1.

He was commenced on empiric intravenous ceftriaxone, as well as supplemental oxygen and nasogastric rehydration. He continued to have spiking fevers after five days of broad-spectrum antibiotic therapy. Serial clinical examinations demonstrated a desquamating perineal rash, peri-oral changes, and cervical lymphadenopathy.

Together with a history of bilateral conjunctival redness prior to hospitalization, these features formed the basis for a likely diagnosis of KD. There were also features of MAS (bicytopenia, hyperferritinaemia, hypertriglyceridaemia, elevated transaminases).

Echocardiography demonstrated dilatation of the left anterior descending coronary artery, (diameter 2.0 mm; Z-score 2.9), and a small pericardial effusion. The child received IVIG 2 g/kg over 12 h on the seventh day of admission, which was followed by defervescence and improvement in serum ferritin and platelet count. He was also commenced on intravenous methylprednisolone 2 mg/kg/day and aspirin 30 mg/kg/day in four divided doses.

**Fig. 8:**
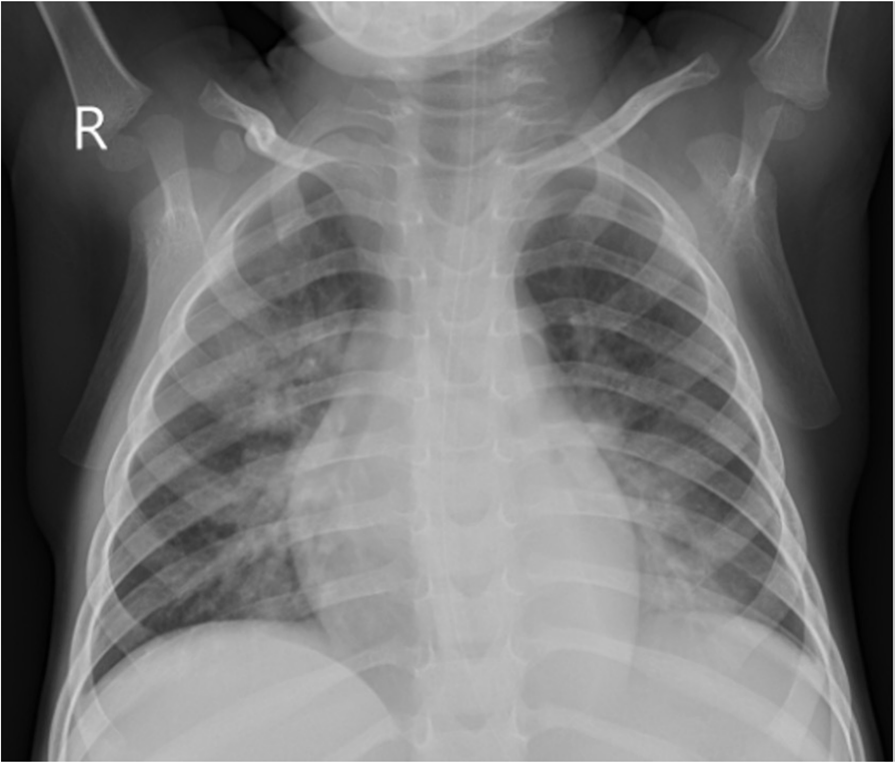
Case 3, Chest radiograph on day 9 of admission. Chest radiograph showing bilateral confluent air space opacification in the bilateral perihilar region, with patchy consolidation in the right upper lobe and left lower lobe

On the ninth day of admission he developed progressive respiratory distress and new foci of patchy consolidation on chest radiograph (Fig. 8), but remained afebrile.

Repeat inflammatory markers demonstrated rising CRP and white cell count, but downward-trending serum ferritin. He required escalation to high-flow nasal cannula oxygen and started second-line antibiotic therapy for presumed hospital-acquired pneumonia with piperacillin-tazobactam and amikacin. Methylprednisolone was discontinued after two days due to a concern of immunosuppression. Repeat blood culture was negative. Clinical improvement was seen with empiric second-line antibiotic and supportive therapy, and he was gradually weaned to room air. Repeat echocardiography demonstrated normal coronary arteries. He was discharged on aspirin 5 mg/kg/day after 15 days in hospital. He was seen for follow-up two weeks later and was noted to be clinically well with low inflammatory markers, recovering anaemia, and rebound thrombocytosis. Repeat echocardiography after six weeks confirmed resolution of the coronary aneurysm and aspirin was discontinued.

### Case 4 (Fig. 9)

**Fig. 9:**
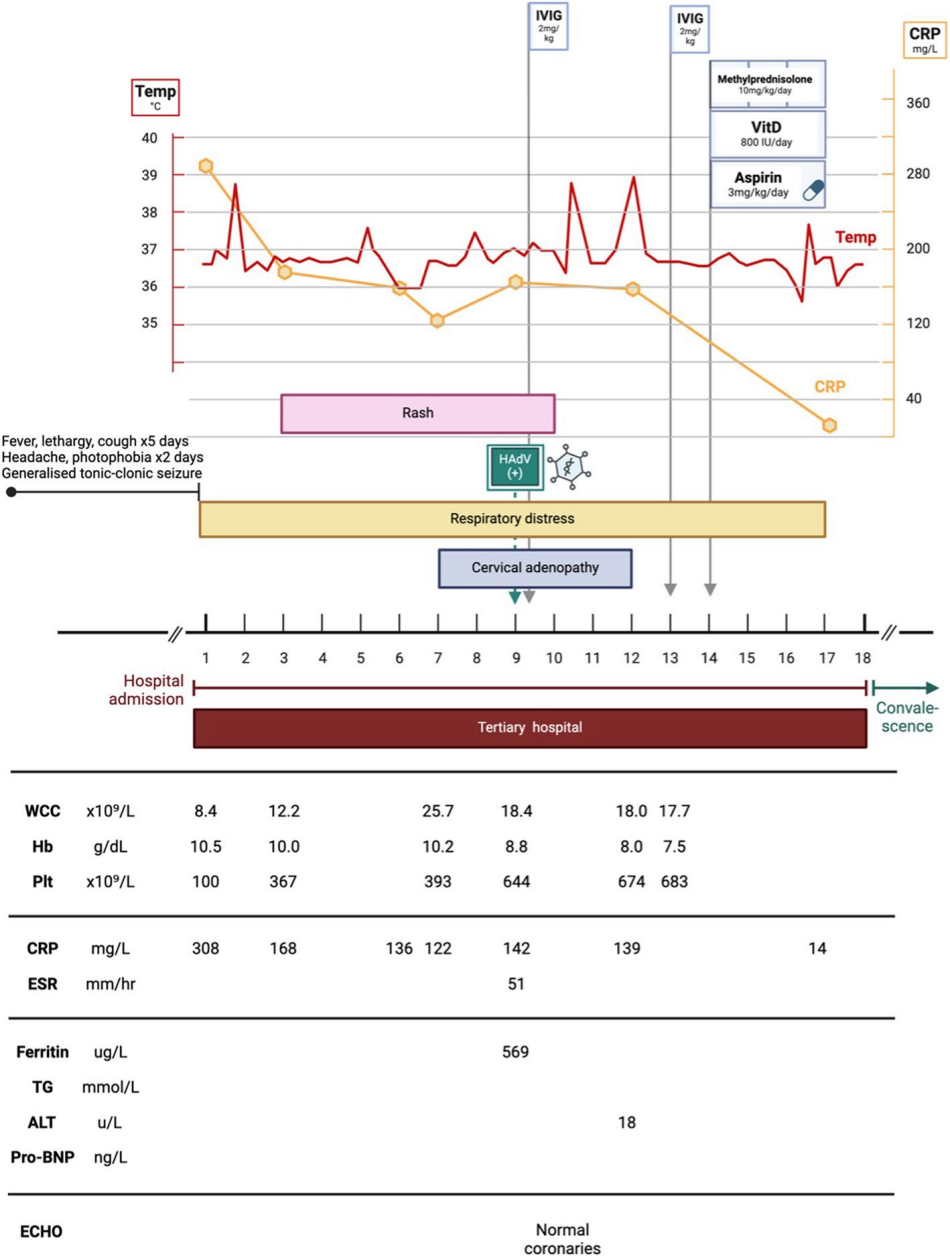
Case 4, Clinical and laboratory features. IVIG (intravenous immune globulin); vitD (vitamin D); HAdV (human adenovirus); WCC (white cell count); Hb (haemoglobin); Plt (platelet count); CRP (C-reactive protein); ESR (erythrocyte sedimentation rate); TG (triglycerides); ALT (alanine transaminase); pro-BNP (pro-brain natriuretic peptide)

A 2-year 9-month-old boy presented to a referral hospital with a seizure, and history of fever, lethargy and cough for five days, and headache and photophobia for two days.

He was immunised according to the national South African schedule, including BCG, but had missed one immunisation (hexavalent DTaP-HBV-IPV-Hib fourth dose) due at 18 months. He had evidence of moderate wasting, with a weight-for-height measurement that plotted between the −2 and −3 *z-score* lines on a standard World Health Organisation growth chart. He was previously well, with no prior hospital admissions and no chronic conditions.

On examination he appeared acutely ill, with respiratory distress and irritability.

Although apyrexial at presentation, he developed high-grade spiking fever in the ward. Chest radiograph revealed hyperinflation and right lung patchy opacities. Laboratory investigations (Fig. 9) demonstrated microcytic anaemia, elevated acute phase reactants, and hyperferritinaemia. HAdV and rhinovirus were detected on nasopharyngeal aspirate PCR. Further work-up is summarised in Table 1.

The child was commenced on empiric intravenous ceftriaxone and acyclovir, humidified oxygen via nasal prongs, and oral paracetamol. Over the following three days, he developed progressive respiratory distress, temperature spikes, and a fine erythematous maculopapular rash over the torso and abdomen. Following a five-day course of ceftriaxone, ongoing temperature spikes and persistently elevated CRP prompted escalation of antibiotic therapy to piperacillin-tazobactam, amikacin, and azithromycin, to cover empirically for hospital-acquired and atypical organisms.

However, repeat blood and urine cultures were sterile. His clinical condition continued to worsen over the next few days, with ongoing fever and irritability, worsening respiratory distress and abdominal pain. The rash became widespread, with erythematous popular lesions involving the trunk and limbs, and associated cervical lymphadenopathy. Repeat chest radiograph showed progressive opacification involving the right upper lobe. He was placed on high flow nasal cannula oxygen and antibiotic therapy was escalated to meropenem.

The child was assessed to have KD based on persistent fever, rash, lymphadenopathy, elevated acute phase reactants, and poor response to empiric antibiotic therapy. His clinical course was further complicated by MAS, as evidenced by hyperferritinaemia, coagulopathy, elevated D-dimers, progressive cytopenias, and multisystem involvement. Echocardiography revealed normal function and excluded coronary aneurysms. A differential diagnosis of MIS-C was considered, however SARS-CoV-2 antigen and antibody testing was negative. On day nine of admission, he received IVIG 2 g/kg over 12 h. Ongoing fever, irritability and abdominal pain prompted the administration of a second IVIG dose on day 13 of hospital stay. He also received aspirin 3 mg/kg/day. A persistently elevated CRP and ongoing fever after two doses of IVIG prompted further treatment with intravenous methylprednisolone 10 mg/kg/day for three days. The response to pulsed intravenous steroid therapy was dramatic, with defervescence following the first methylprednisolone dose, and resolution of respiratory symptoms, abdominal pain, and rash over the following few days. Oxygen therapy was discontinued and CRP normalised. The child was discharged after 18 days in hospital.

At follow up two weeks later, he was clinically well with a low CRP, improving anaemia, and rebound thrombocytosis.

## Discussion and conclusions

KD and acute adenoviral infection may be clinically indistinguishable, due to shared features of prodromal respiratory and/or gastrointestinal symptoms, prolonged fever, mucocutaneous inflammation, and elevated acute phase reactants. Desquamating rash, BCG scar erythema with induration, rebound thrombocytosis and coronary artery dilatation are relatively specific indicators of KD, but do not appear in every case and may be absent in the acute phase, when timely diagnosis and treatment is crucial to prevent CAA. Furthermore, simultaneous HAdV infection and KD appears to be common.

Adenoviruses are common human pathogens. Children are typically serially infected with multiple different adenovirus types in the first years of life. Historically, detection of adenoviral infection precluded a diagnosis of KD. However, multiple cases of children with HAdV infection and specific features of KD have been reported in the literature [10, 14–16]. The significance of HAdV infection in the setting of KD remains unclear. Asymptomatic adenoviral infection is common in children [17]. Additionally, latent HAdV infection may be followed by reactivation and low-level viral shedding in response to inflammatory stimuli [18, 19]. Recent population reports describe a temporal association between HAdV infections and KD incidence, suggesting that HAdV infection may contribute to KD immunopathogenesis in some cases [1, 2]. A population-based cohort study conducted in Taiwan reported a five-fold greater risk of KD in children with previous HAdV infection compared to controls [20]. A population-based time series analysis conducted in France estimated 24% of KD cases to be potentially attributable to HAdV infection [1]. Although clinical and epidemiological data supporting an association between HAdV and KD exist, experimental evidence of a role for HAdV in the immunopathogenesis of KD is lacking.

The epidemiology of KD is consistent with an infectious cause, particularly a respiratory virus [21]. KD has been associated with epidemics in Japan, the United States, and Europe, with geographic spread similar to airborne viral epidemics [22–25]. KD incidence typically peaks in winter/spring, mirroring seasonal respiratory viruses [26].

During the COVID pandemic of 2019–2020, control measures designed to limit the spread of respiratory viruses were imposed across most regions of the world, and were followed spatially and temporally by dramatic declines in KD incidence [27–30]. Associations between KD and numerous infectious triggers have been proposed, but despite significant research efforts no individual pathogen has been conclusively demonstrated to cause KD [21, 31, 32]. These data support the hypothesis that KD is probably caused by exposure to a common seasonal respiratory virus, or group of viruses, in genetically predisposed children. Recent work has demonstrated antibodies in some children with Kawasaki, that localise to antigens located in inclusion bodies bronchial epithelial cells, further hinting at a potential single pathogenic trigger in KD [33].

Several case reports of KD and concurrent HAdV have been published [10, 14–16]. Additionally, cases of an hyperinflammatory syndrome associated with HAdV have also been described [34–36]. A retrospective study conducted in the United States compared KD patients with and without concurrent respiratory viral infection [14]. This study reported significantly higher frequency of coronary artery involvement in children with KD and any concurrent viral infection (42%), compared to children with only KD (14%, p = 0.02). CAA rate was greatest in KD cases with HAdV infection (66%). Notably, this observation was independent of the timing of IVIG administration after onset of fever.

There were no other significant differences in clinical features or outcomes between subgroups.

A case report from India described an infant with HAdV infection and features of a hyperinflammatory syndrome, complicated with CAA and MAS [15]. The child developed persistent fever, multiorgan involvement, and elevated acute phase reactants following a brief respiratory prodrome. Echocardiography demonstrated left main CAA. HAdV was detected on nasopharyngeal aspirate PCR and SARS-CoV-2 IgG antibodies were positive. Similarly to Case 2 described above, this patient presented a diagnostic dilemma as diagnostic criteria for KD, MIS-C, and MAS were fulfilled. The managingclinicians ultimately chose to treat the patient with IVIG, but to withhold steroid therapy and aspirin. The infant responded well to multi-dose IVIG therapy.

A case series from China reported a case of KD complicated by MAS, coronary artery involvement and IVIG resistance, who tested HAdV-positive on bronchoalveolar lavage specimen [10]. Another case report described an infant with HAdV infection and KD, complicated by fatal coronary arteritis [16]. Multiple CAA and thromboses were identified at autopsy, and viral culture of lymph node specimens was HAdV-positive.

We have described four cases of concurrent adenoviral infection and complicated KD.

These cases presented a diagnostic challenge to the managing clinicians and are illustrative of the challenges of distinguishing between acute infections and KD, and managing KD with concurrent infection. An interesting unifying feature of the cases was the marked severity of KD, with all four children presenting with severe KD and features of MAS. Two patients had IVIG resistance, with recrudescent fever more than 36 h after initial IVIG therapy, and two children developed coronary artery abnormalities.

Early diagnosis of KD was established in all cases, facilitating appropriate IVIG therapy and good outcomes. However, we note a delay of several days between MAS diagnosis and use of high-dose intravenous steroid therapy. We postulate that clinicians’ concerns about steroid immunosuppression in patients with active infection likely contributed to these delays. In these cases, use of intravenous steroids was associated with substantial clinical improvement and rapid resolution of MAS. All four children were discharged after a few weeks, and disease resolution was confirmed at follow up in three of four cases. One patient was lost to follow up.

The existing literature clearly establishes that KD and HAdV infection can occur concurrently. Furthermore, and in keeping with our experience of four cases of simultaneous KD and HAdV infection, existing reports suggest a more severe clinical phenotype of KD in this setting, with higher rates of complications, including CAA and MAS. We postulate that HAdV infection may trigger immune dysregulation in at-risk children, resulting in a hyperinflammatory syndrome which is clinically consistent with KD and MAS. It is also possible that cases of KD and concurrent HAdV infection with a less severe clinical phenotype are not reported, due to under-diagnosis of KD with milder features, and non-detection of HAdV infection when viral testing is not performed.

Further prospective research is required to investigate the relationship between KD and concurrent HAdV infection, to define the clinical phenotype of simultaneous KD and HAdV infection, and to explore a possible role for HAdV in the immunopathogenesis of KD.

## Data Availability

The dataset generated and analysed during the current study is not publicly available, as it contains personal demographic and health information of the participants. A de-identified subset of the data used during the study is available from the corresponding author on reasonable request, with the permission of Dr K. Webb and Dr L. Githinji.

## Abbreviations

BCG: Bacillus Calmette-Guérin
BMAT: Bone marrow aspirate/trephine
CAA: Coronary artery aneurysm
CSF: Cerebrospinal fluid
CPAP: Continuous positive airway pressure
CRP: C-reactive protein
Ct: Cycle threshold
DTaP-HBV-IPV-Hib: Diphtheria, Tetanus, acellular Pertussis / Hepatitis B Inactivated Poliovirus/Haemophilus influenzae type b
ESBL: Extended-spectrum beta lactamase
FiO2: Fraction of inspired oxygen
HAdV: Human adenovirus
HIV: Human immunodeficiency virus
IgG-N: Immunoglobulin G nucleocapsid
ICU: Intensive care unit
IVIG: Intravenous immunoglobulin
KD: Kawasaki disease
MAS: Macrophage activation syndrome
MIS-C: Multisystem inflammatory syndrome in children
PCR: Polymerase chain reaction
SARS-CoV-2: Severe acute respiratory syndrome related to coronavirus-2
